# Finding long protein products of alternatively spliced genes

**DOI:** 10.1186/1471-2105-13-S12-A12

**Published:** 2012-07-31

**Authors:** Neil Moore, Jerzy W Jaromczyk, Christopher L Schardl

**Affiliations:** 1 Department of Computer Science, University of Kentucky, Lexington, KY 40506, USA; 2 Department of Plant Pathology, University of Kentucky, Lexington, KY 40506, USA

## Background

In eukaryotes, pre-mRNA molecules undergo splicing, which is the removal of sequences called introns to produce mature mRNA transcripts whose open reading frames (ORFs) may then be translated to proteins. Often this splicing step may be performed in many ways - a situation known as alternative splicing [1,2] that can be described by structures such as splice graphs [3]. Alternative splicings, even those that differ only slightly, may result in proteins with substantially different biological properties [4,5].

## Materials and methods

We have developed an algorithm for finding the longest ORFs of alternatively spliced transcripts described by splice graphs. Our algorithm executes in time linear in the size of the splice graph (and therefore optimal), determining the splicings that result in an open reading frame encoding a maximal-length protein for that gene. We show how our algorithm may be used to help identify biologically interesting protein products from RNA-seq data.
